# Why am I obsessed with viewing mukbang ASMR? The roles of mediated voyeurism and intertemporal choice

**DOI:** 10.1371/journal.pone.0308549

**Published:** 2024-09-19

**Authors:** Nan Jiang, Kok Wei Khong, Mobai Chen, Kim Leng Khoo, Jesrina Ann Xavier, Manimekalai Jambulingam

**Affiliations:** 1 Faculty of Business and Law, Taylor’s University, Selangor, Malaysia; 2 Business School, University of Nottingham, Ningbo, Zhejiang, China; 3 School of Media, Qingdao University of Science and Technology, Qingdao, Shandong, China; 4 School of Journalism and Communication, Shandong University, Jinan, Shandong, China; 5 Sunway Business School, Sunway University, Sunway City, Malaysia; Shaanxi Provincial People’s Hospital, CHINA

## Abstract

This study investigates the determinants of the obsessive view of mukbang autonomous sensory meridian response (Mukbang ASMR) and examines the moderation role of intertemporal choice and the mediating effect of mediated voyeurism among university students in Malaysia. A quantitative survey was conducted with 408 university students in Malaysia who viewed mukbang channel(s) often on social media. PLS-SEM is adopted to examine the associated paths and effects. The results demonstrate the significant impact of alienation and novelty, vicarious satisfaction, companionship and loneliness on mukbang ASMR obsession. Mediated voyeurism intervenes the effects of alienation and novelty on mukbang ASMR and intertemporal choice positively moderates the relationship between companionship and loneliness and mukbang ASMR. This study constructs a model to estimate Mukbang ASMR obsession by identifying specific motives and relationships among key factors, highlighting loneliness as the most effective determinant of mukbang ASMR among Malaysian younger generation. Research results provide an extended understanding of the mukbang ASMR, offering valuable insights in the areas of lifestyle, social well-being, and social media consumption.

## Introduction

Nowadays, digital media consumption and social media usage have become dominant entertainment activities in people’s daily life. Driven by increased mobile device usage, digital media consumption has penetrated across all regions. Up to April 2024, there were 5.17 billion digital media users globally, which occupies more than 60% of the world population. And this number is expected to increase up to 6 million worldwide users by 2018 [1]. Different from subscription-based digital media consumption (e.g. Netflix, Amazon Prime), social media consumption, especially social media platforms enable individuals to create, search, interact, and share their own content. Video-based social media (e.g. YouTube, TikTok) and image-based social media (e.g Facebook, Instagram, and Weibo) become more popular. In addition, food-related social media content, such as mukbang ASMR or eating broadcasting attracts more younger generation’s attention. Mukbang ASMR (also known as eating broadcasting) emerged and became popular worldwide. Billions of subscribers head to YouTube and Instagram just to watch other people preparing food and broadcasting their eating ASMR. The word mukbang is a portmanteau of the South Korean words for ‘eating’ (meokneun) and ‘broadcast’ (bangsong). It emerged in South Korea in 2014 and began gaining interest in Malaysia since 2018 [2]. This trend has been expedited since the Covid pandemic and has become a trend for the younger generation. Some local mukbangers have gained hundreds of thousands of followers on YouTube, TikTok, Facebook, and Instagram. It offers entertainment and gratification but also leads to harmful consequences. Mukbang ASMR changes people’s minds about food and impacts their behaviour. Nowadays, some university students consider mukbang ASMR to be a low-barrier entry startup career or a part-time job opportunity. Many enjoy the instant fun of mukbang but overlook the negative and underlying long-term consequences on health, lifestyle, and social well-being. The existing research on mukbang ASMR is relatively limited and primarily restricted to adolescents or youths (e.g. high school) in the regions of South Korea, the United Kingdom, and the United States. Existing literature mainly emphasizes the impact of Mukbang on potential problematic behaviour (e.g. binge eating, food choice and practice, social media addiction), and associated consequences (e.g. internet addiction, eating disorder, obesity, and consumption norms). Existing studies and literature have often overemphasized the negative impact of mukbang ASMR, citing issues such as internet addiction, binge eating, and obesity. Logically, the more addicted viewers are to mukbang, the more likely they are to engage in binge eating or develop obesity. However, even excessive viewing of mukbang ASMR can be problematic with negative consequences, these impacts might not be classified as addiction [3, 4] for the following reasons: (i) Mukbang ASMR involves non-chemical interactions between viewers and social media, distinguishing it from drug addiction; (ii) There is a lack of precise medical or psychological measurements to prove that these viewers are clinically addicted; (iii) Participants in existing studies vary in terms of age, gender, and regions, making it difficult to achieve consistent conclusions. Therefore, while acknowledging the highlighted harmful consequences, this study categorizes mukbang ASMR as a type of problematic behavior [5], rather than an addiction as defined in the medical field. In contrast to definition and category, the fundamental rationale behind mukbang obsession is the essential root of addressing the consequential problematic behaviors. There is limited empirical study to examine Mukbang phenomenon from psychological characteristics in Malaysia within the context of social media consumption. Hence this study investigates the specific motives of Mukbang ASMR obsession from a younger adult’s perspective. This study consolidates existing literature of mukbang ASMR, adopts the Theory of Problem Behaviour from social-psychological perspective, Use & Gratification (UG) and Compensatory Internet Use Models (CIUM) [6, 7] from a media usage perspective. This research aims to answer the following three research questions: (i) What antecedents influence students to keep watching Mukbang ASMR on social media? (ii) Does the younger generation prioritize instant gratification (e.g., Mukbang enjoyment) over long-term health and social well-being? New insights may emerge and enhance the existing knowledge and understanding of Mukbang ASMR.

## Materials

### Social media consumption and mukbang ASMR

As a specific form of digital media consumption, viewing, exploring and sharing content on social media become prevalent. More than 60% world’s population is using social media daily [8]. Social media can be further categorized into different types, including social networking (e.g. Facebook, Weibo), video sharing (e.g. YouTube, TikTok), professional networking (e.g. LinkedIn), and personal blogs. Recently, eating-focused or food-related images and videos have emerged drastically in many social networks (e.g. Instagram, Facebook, and YouTube), resulting in obsessive engagement in social media consumption [9, 10]. Mukbang and ‘Food Porn’ are typical forms of live-stream food broadcasting where channel hosts (mukbangers) consume a large amount of food while interacting with their audience. Additionally, extreme-quality microphones are often utilized to catch the sounds made as an outcome of eating (e.g. crunching sounds) or as an outcome of opening food bundling. These sounds are known as ASMR components, which are perceived to deliver relaxation, a narcotic sensation for certain viewers [11]. The sensorial experience from the hearable and visual improvements of the videos appears to assist viewers with vicariously satisfying mukbang [12]. Mukbang videos are frequently portrayed by their hyper-obsession with the visual and audial encounter of eating food. For instance, mukbangers may move their camera closely to show the food’s pictures and texture, as well as increase amplifier settings to let audiences able to hear the sounds of mastication. Mukbang is categorised into five typologies [13]: (i) ‘Big food fighter/eater king’ with a huge physical body and often participates in food consumption challenges; (ii) ‘calm eater’ with just concentrates on eating instead of raising a ruckus on camera. They endeavour no outrageous challenges. However, they do eat enormous quantities of food nicely and with incredible pleasure; (iii) ‘Weirdo’, who behaves in unusual ways in the Mukbang videos. For instance, grappling with a huge octopus while cooking or popping corn in a griddle to grab the incredible attention of viewers; (iv) ‘Cook’, where they eat the food that cooks by themselves and will describe the recipes to the viewers; (v) ‘Pretty girl/boy’, this kind of host generally dresses up and shows their sexy outfit and slim body and sets up the lighting effects to make their facial composition look great. Mukbangers of this typology normally keep interacting with their followers during live streaming to gain virtual gifts or currency (from their audience).

Embedded with ASMR sensations of relaxation and prosperity, mukbang delivers a temporary sense of gratification or perceived social compensation to audiences. However, it may mislead the audience’s mind, and consequently trigger problematic behaviour and unhealthy social well-being. Although some studies highlight the beneficial impacts of Mukbang, such as entertaining and diminishing people’s perception of stress [14], loneliness [15], social isolation [16], and anxiety [17], most literature focuses its harmful consequences including social media addiction [18], eating disorder [19], detrimental social well-being [20], obesity [21], and sexual abuse motivation [15]. However, even when addressing the same type of consequences of Mukbang (e.g., positive or negative consequences), there are inconsistencies in the study findings. These inconsistencies may stem from differences in research design (qualitative vs. quantitative methods), diverse research focuses (e.g., psychological vs. communication perspectives), or participant demographics (e.g., adolescents, children, female channel hosts, and individuals on diets). A cross-cultural findings of Mukbang’s key dimensions are extracted from a literature scoping (S2 Appendix).

From a sociological perspective, mukbang ASMR viewing obsession counts as a kind of risk engagement whose determinants are primarily reflected by the three interrelated clusters of problem behaviour theory: personality, environment, and behaviour. Problematic behaviour is defined as ‘behaviour that is socially defined as a problem, a source of concern, or as undesirable by the norms of conventional society … and its occurrence usually elicits some kind of social control response [5]. Problem behaviour theory predicts a variety of risk engagement, including drinking, drug use, smoking, sexual intercourse, and delinquent behaviur [22]. Most present studies investigate the problem behaviour at relatively younger age levels, such as adolescents or college students. With the same token, Mukbang ASMR could well constitute a similar ‘syndrome’ of problematic behaviour. Such ‘syndrome’ is portrayed as ‘food porn’ in live streaming food broadcasting. However, food porn was initially defined as an academic and intellectual concept in the context of aesthetics studies by Michael Jacobson in 1977. Food pron is used to describe a type of ‘glossy, art image of often high-end food’ [23]. Such beautiful food often embodies the essence of cultural distinction, unattainability and elevated taste [24]. Audiences can almost ‘feel’ its beauty and deliciousness through the screens. While, in the digital age, food porn develops into a kind of trendy hashtag in social media (e.g. Instagram). The definition and understanding of food pron are turning into controversial transgression [24, 25] and are often associated with sensuality, gender, and body, especially when the channel host is a comely and sexy female mukbanger. The food porn notion transfers from an academic and aesthetic visual into a daily used term of ‘mouth-watering’ [26] and ‘inaccessible’. The perception and interpretation mainly depend on the particular context and not every food presented in social media can be considered as food porn. Food porn focuses on the pleasant and sensual dimension of food-related content [27] and provides audiences with vicarious entertainment [28], especially when they receive stimulation toward the sensation of sight, touch, taste, smell, and hearing provided by the picture and sound of eating [16, 29]. Besides these food-oriented cognitive stimulations, other sensation-seeking traits could tap the tendency of seeking varied, novel, and intense experiences from different social dimensions, such as vicarious consumption, mediated voyeurism, alienation seeking, and perceived loneliness. Consequently, a risk behaviour: mukbang ASMR obsession could be formed and lead to social well-being concerns and health issues. And the building and development of such problematic behaviur could be further boosted or neglected by viewers’ time perspective notion: the intertemporal choice. Intertemporal choice is an economic theory also known as the intertemporal concept that involves deciding between smaller, sooner and larger, later rewards [30]. Intertemporal choice addresses the time perspective notion suggesting that people who evaluate future outcomes as less important and far from happening are more likely to sacrifice that future for the sooner joy [31]. It is the process by which people make decisions about what and how much to do at various points in time when choices at one time influence the possibilities available at other points in time [32]. These choices are influenced by the relevant value people assign to two or more payoffs at different points in time. Therefore, if the audiences perceive the current and instant fun (e.g., mukbang joy) as more worthwhile than their future health and social well-being, then such problematic behaviour of Mukbang ASMR obsession is more likely to form and vice verse.

**Fig 1:**
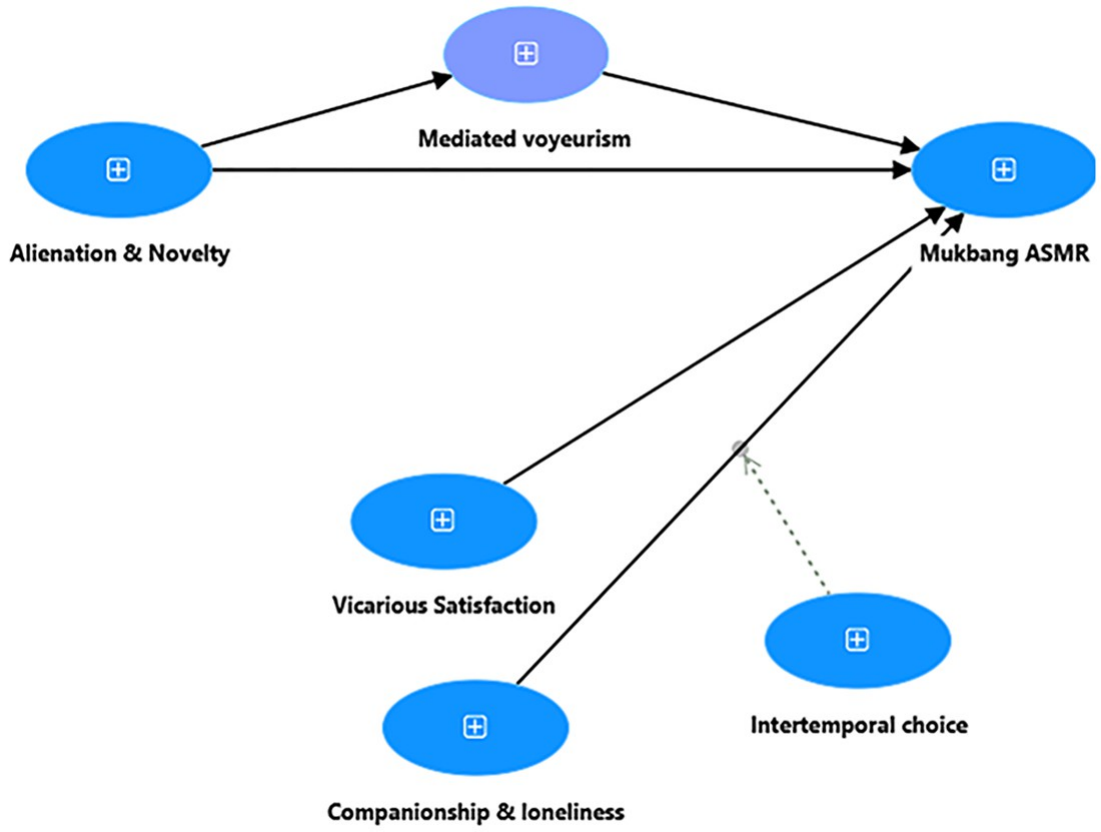
Mukbang ASMR conceptual framework.

From digital media and usage perspectives, the Use & Gratification (UG) is a typical model in the area of media and communication that evaluates an individual’s motivation in media access and usage [7]. According to UG, users of media are motivated by two different types of gratifications: gratifications sought and gratifications obtained. Gratifications sought refer to users’ expectations of the types and content of gratifications they would get from using media, whereas gratifications obtained refer to the needs satisfied by media use [33]. UG theory is transferrable to Mukang ASMR and food broadcasting. Mukbang ASMR is also a type of consumption behaviour where a sense of happiness and relief can be experienced from live-stream broadcasting, such as consumption noise, cooking sounds, and facial expressions. Unlike general and traditional digital media consumption, Mukbang ASMR audiences can experience three distinct types of gratification: (i) the enjoyment of viewing Mukbang (ii) joy of interacting with Mukbangers or other viewers, and (iii) the sensory amusement from ASMR sounds (i.g. eating or cooking noises). Although gratification plays a significant role in Mukbang ASMR, the UG theory does not comprehensively explain the Mukbang phenomenon in the context of digital media consumption, mainly due to the following reasons: (i) UG overemphasizes gratification and exaggerates the joy effect between media and audience, but overlooks the impact of other perspectives (e.g. loneliness, mediated voyeurism, and body image concern) in Mukang obsession; (ii) Initially, UG theory was developed and applied in traditional media channels (e.g. TV, DVD, paper magazines). The gratification impact may not remain as the dominant antecedent in today’s digital media landscape; (iii) UG is more useful in describing Mukbang ASMR, but lacks predictive capability in assessing the gratification effect in social media consumption, excessive usage or problematic behaviour. Apart from gratifications, the compensatory internet use model (CIUM) states that people’s various internet engagements are mainly due to their unattained offline needs [34]. Although CIUM focuses on individual’s psychological desires, not every deficiency need can be satisfied through internet usage. CIUM sounds too general in guiding people’s online behaviour where most suppressed desires (unattained needs) turn into a ‘one-size-fits-all’ answer for various internet activities. CIUM oversimplifies the complex interplay between humans and internet. It also neglects many positive and non-compensation-oriented motives in internet usage. For instance, Mukbang ASMR audiences may not always seek compensation due to negative perceptions (e.g. loneliness or stress) or unmet desires. Instead, they may simply enjoy the immediate fun, novelty or vicarious consumption provided by watching Mukang broadcasting. Therefore, this study aims to offer comprehensive insights into Mukbang ASMR by integrating both Uses and Gratifications (UG) and Compensatory Internet Use Model (CIUM) from media usage and social-psychological perspectives. The proposed Mukbang ASMR model is presented in Fig 1.

### Vicarious satisfaction

Vicarious satisfaction (also known as vicarious eating or vicarious consumption) is one of the main motivations of mukbang ASMR viewing obsession, especially for those followers (viewers) who are on diets, who love food, and who want to obtain virtual satisfaction from watching the consumption of a wide range of different food in mukbang live streaming videos [23, 35]. Watching mukbang appears to help such individuals satisfy food cravings, experience the feeling of binge eating themselves, and have vicarious satisfaction via visual and audio stimulation [36]. Vicarious satisfaction serves as compensation for acts that an individual would not be able to perform in real life and/or as a fulfillment of known experiences regarding the watched act via triggering a memory [6, 37]. Some scholars define vicarious satisfaction as a relieved perception of certain physical and/or psychological desires that may not be performed at the current moment. Viewers have been reported to achieve vicarious satisfaction from different online activities, including viewing fetish-themed pornography movies [38] and online dating [39]. Online gaming and gambling have also been reported to be preferred as a leisure activity by providing vicarious satisfaction of making the impossible appear possible [15, 40]. Similarly, Mukbang ASMR fulfills the audiences’ need for virtual satisfaction and compensation. Mukbang offers viewers a kind of vicarious pleasure, making them feel as though they are eating the food themselves [41] When watching Mukbang ASMR, audiences experience the sensation of consuming the same food, with the taste imagined in their minds rather than through physical chewing or swallowing. This vicarious satisfaction reflects the ’unattained’ desire described in the Compensatory Internet Use Model (CIUM) theory, where viewers seek to compensate for such unmet needs by obsessively watching Mukbang ASMR. Research has highlighted vicarious satisfaction as a positive effect in the areas of body image, dieting, and weight management. The primary beneficiaries are females on diets, who may be less concerned with the Mukbanger’s appearance or attire and more focused on the appetizing food, finding it more appealing and attractive [35]. Viewers seek vicarious satisfaction from viewing the food being consumed, where the vicarious pleasure comes from the eating performance of the mukbanger in terms of food nature (healthy vs unhealthy), food selection (e.g., viewers’ desired dishes), eating manner, consumption quantity, sounds of eating, and food appearance. In this process, viewers feel as if they are chewing the same food or as if they are the Mukbangers eating the food in the video, consequently perceiving satiety and pleasure. This sense of satiety and vicarious satisfaction can be increased if the Mukbanger is eating food that is desired or requested by the audience.

**H1**: Vicarious consumption positively influences mukbang ASMR.

### Alienation and novelty

Perceived alienation and novelty refer to the degree of feeling that the content is new and fresh and how new a certain content is compared to an existing one [42]. It depends on whether differences are occurring between the existing and new ones [43]. Audiences tend to view mukbang ASMR videos because it is unique and food-related. Watching the video is ‘sake for alienation and novel’, which can bring them happiness and feel excited. Mukbang ASMR obscures public and private in customary spaces and uncommon food varieties and eating exhibitions. From a more extensive perspective, audiences tend to watch unscripted content, such as live-stream food broadcasting because they consider such shows as a source to peep into the existence of genuine and realistic characters. Unscripted shows with content food such as reality food shows, mukbang live-streaming, and eating video logs (vlogs), as a form of presenting and sharing regular life activities, which is perceived as novelty and alienation. Audiences who are novelty-seeker will positively connect themselves to the content of mukbang ASMR videos [44]. When people are continuously looking for alienation and novelty stimulus [45], their impression of that stimulus depends on whether it is seen to be valuable, new, unique or even unsafe, which in fact, decides if they acknowledge or ignore such stimulus. The remarkable nature and performance of mukbang ASMR will fulfill the novelty-seekers who seek alienation of content in social media. Hence, alienation and novelty could be another determinant of mukbang ASMR obsession.

**H2**: Alienation and novelty positively influence mukbang ASMR

### Companionship and loneliness and intertemporal choice

Loneliness is a typical human feeling of social isolation that has been related to several detrimental physical and mental health concerns [46]. Mukbang ASMR can help alleviate viewers’ feeling of loneliness and encourage social interaction with the channel host (mukbanger) and other viewers via virtual platforms [42, 47]. On the par with the increasing number of single households in South Korea, loneliness and companionship are identified as one of the primary factors why people are obsessed with mukbang ASMR [13, 48]. Not just in South Korea, with Asia communal food culture, people are keen to dine together, sharing tables and dishes. It is rooted in shared commensality [49], the social practice of sharing food and eating together with families or friends. However, as the number increased individual mobility, most university students and working adults barely live together with their families [50]. Eating together is becoming a precious experience nowadays, while the perceived loneliness and desire for companionship have increased dramatically. While mukbang ASMR alludes to human co-dependency and reciprocal commitment through a virtual meal observation. Some audiences even eat their own meals in front of computer screens together with the mukbangers on camera to enjoy the personal and collective mealtime. Mukbang ASMR assists lonely individuals to feel more connected and less socially isolated. In addition, audiences who perceive lonely or isolated are more eager to seek companionship and, hence may be more likely to value the instant companion and intend to trade off immediate joy with future health and social well-being, although they are aware of the associated consequences. The intertemporal choice primarily depends on which component is more valuable in decision-making [32, 51]. Lonely viewers may prefer smaller and sooner rewards (instant relief/pleasure) rather than larger and future rewards (e.g., healthy lifestyle). The key decision is whether it is worthy to trade off today’s joy with future rewards. Which one is more valuable: now or the future? Should we value current joy (mukbang ASMR) over social well-being and future health? It does not have much to do with self-discipline or rational thinking. Socially isolated and susceptible individuals are more likely to scarify their future lifestyle for sooner relief or gratification.

**H3**: Companionship and loneliness positively influence mukbang ASMR.
**H5**: Intertemporal choice moderates the relationship between loneliness and mukbang ASMR.

### Mediated voyeurism

Mediated voyeurism is the consumption of uncovering scenes and data about others’ real and unprotectable lives, mostly not for entertainment purposes but rather habitually to the detriment of privacy and security, through social media or network broadcast channels [52, 53]. Mediated voyeurism alludes to the propensity to notice or ‘peep’ into the living routine of someone without their cognizant information [15]. The voyeur partakes in a covert action of watching someone as this observation can provide the voyeur with holding power clandestinely over the target by empowering the voyeur to take part in an uneven progression of data as the target does not know that he/she is being observed [54, 55]. This longing to see the lifestyle and activities of others is ‘mediated’ by the network channel through the broadcasted activities of a host on social media. In mukbang ASMR live stream, the mukbanger always repeats the subtleties of their day-to-day routines, thus, allowing viewers to acquire knowledge into the mukbanger’s personal lives, which is crude and unscripted, therefore fulfilling viewers’ voyeuristic longings [55]. Viewers may perceive such ‘peeped’ content as alienation and novelty, especially for female mukbangers with cute faces or sexy outfits. Mukbang might be comprehended as fetishizing women binge eating [56], where self-portrayal of women eating huge amounts of harmful or unhealthy food showing the ‘*shameful appetite*’ (p.784) that women conceal which was susceptible to sexualising women’s bodies [16]. The reinforcement of normative values of thinness and consumerism could be associated with the potential sexual objectification of the female body [56]. With societal norms [57], females are often expected to embody feminine traits, maintain a slim physique, and behave appropriately, such as being gentle, eating slowly and quietly, taking care of kids at home, and presenting a decent appearance. It is uncommon to see females consuming enormous amounts of food or eating in a manner deemed unappealing in public, such as loud chewing and slurping, messy eating, excessive large bites, etc. Mukbang ASMR fills people’s desire for mediated voyeurism by offering a glimpse into ’authentic’ and ’unscripted’ live-stream eating shows. This sense of voyeurism can be stronger when the female mukbanger eats food requested by male viewers or receives virtual currency from them. Some viewers neither care about the mukbangers nor the food but consider them as ‘virtual prostitutes’ who consume whatever viewers desire in exchange for actual bonuses or virtual currency [35]. However, the condition described above assumes that mukbangers are paid by viewers or receive monetary incentives from the viewers who may sexually interpret the mukbang shows. However, in most cases, mukbangers bear the expense of food and materials. Their main revenue streams from audience flow and social media platform advertisements. It is inappropriate to portray female Mukbangers as "prostitutes" based on the viewer’s individual mediated voyeurism. Mukbang live-stream broadcasting neither includes pornography performances (e.g., adult videos or clips), nor results in any pornographic consequences, such as diminishing sexual responses toward real-life partners or leading to abnormal sexual behaviors. Instead, it does have a certain positive impact on people’s social life, such as enjoyment, and relief of stress and anxiety. In live-stream food broadcasting, the food plays an important role and the effect of mediated voyeurism does not directly impact mukbang ASMR, but mediates the perceived alienation and novelty and indirectly influences mukbang ASMR. If the effect of mediated voyeurism were stronger enough, then the obsessive viewing content would be pornography shows rather than mukbang ASMR food broadcasting, hence:

**H4:** Mediated voyeurism mediates the relationship between alienation and novelty and mukbang ASMR

## Method

This study uses partial least squares structural equation modeling (PLS-SEM) for data analysis. PLS-SEM offers comprehensive capabilities for exploring research, analyzing causal relationships, and testing theoretical models [58]. Data analysis begins with measurement model assessment and is followed by structural model examination. The survey measurement scale was developed and modified from relevant previous studies. The reliability consistency and factor loading of each latent variable are presented in S1 Appendix. Few preliminary questions were set to screen the participants and ensure only those who have subscribed mukbang ASMR channel or often viewed mukbang ASMR videos are counted. Those participants who indicated they rarely view mukbang ASMR or are not interested in food broadcasting are eliminated from the sample selection. The bulletin boards of three university common core modules are used to accommodate data collection over four weeks (8^th^ October to 10^th^ November 2022). The survey link was posted on each module’s bulletin board to maximize the response rate from students. Aiming to increase awareness and participation, an incentive of 30 tokens was offered, with a chance to win petrol gift vouchers worth RM50 (USD 11). YouTube and Instagram are the primary social media platforms mentioned in the survey link. This study complies with ethical standards by ensuring that all participants were fully informed about the nature and purpose of the research, evidenced by the written informed consent provided on the first page of the online distributed questionnaire. Each participant has the autonomy to opt out of this survey at any given moment, safeguarding their right to privacy. Measures were in place to ensure the confidentiality of participant data, in compliance with the Personal Data Protection Act of 2010. Research ethics approval was granted by the Human Ethics Committee (HEC) from the authors’ university.

The pilot test gathered 56 student samples to assess the reliability and validity of the measurement instruments. Measurement indicators that do not accurately measure the intended constructs are removed. Data with missing values or response errors (e.g. identical responses across multiple questions) are excluded too. The measurement scale of intertemporal choice [32] is also converted to a 7-point Likert scale to ensure consistency in measurement and analysis. The number of parameters is recommended in estimating the sample size of confirmatory factor analysis (CFA) [59], where the ideal size-to-parameter ratio of 20:1 (N:q) in the structural equation model (SEM) is recommended [60]. Effect size [61] and G-power are adopted in estimating the minimum required sample size in this study. Except for intertemporal choice (moderator), four predictors (including mediator) are counted in estimating the minimum required sample size. With G-Power liner multiple regression fixed model (R² deviation from zero), the calculated minimum sample size is 356 (effect size f² = 0.034, σ = 0.05, power (1-ᵦ error prob) = 0.80, number of predictors = 4). A total of 408 out of 672 received surveys are valid samples and the collected sample is sufficient for SEM modeling. A demographic description of respondents is presented in Table 1.

**Table 1:** Demographic description of respondents.

	Yes	No
Do you subscribe/follow any Mukbang channel on social media?	74.51%	25.49%
Do you view Mukbang ASMR often?	70.01%	29.90%
Are you aware of the potential consequence of Mukbang ASMR?	87.50%	12.50%
Are you interested in becoming a part-time Mukbanger?	38.24%	61.76%
Do you often eat together with Mukbanger in front of a screen?	11.76%	88.24%
Do you often forward Mukbang videos to your friends or peers?	68.63%	31.37%
Do you like the food consumed by Mukbangers in the video?	70.01%	29.90%
Gender	Male 35.54%	Female 64.46%

## Results and discussion

Convergent validity is used to assess the internal consistency among a set of construct indicators. In the measurement model, both factor loadings and composite reliability (CR) exceed 0.7, and all indicator loading estimates are significant (p<0.001). Average variance extracted (AVE) estimates range from 0.620 to 0.870. The maximum correlation among latent variables is 0.755. The square root of average variance extracted (AVE) for each construct is greater than any correlation between that construct and any other constructs (Table 2). Multicollinearity is further tested by heterotrait-monotrait (HTMT, Table 3) and variance inflation factor (VIF). All HTMT ratios are below 0.85 [62], and the inner model variance inflation factor (VIF) ranges between 1.383 and 3.922. This study meets the criteria for convergent and discriminant validity and the probability of Type II error is low.

**Table 2:** Convergent and discriminant validity (n = 408).

	Cronbach’s alpha	C.R	AVE	AN	CL	IC	MV	M	VS
AN	0.949	0.965	0.71	**0.843**					
CL	0.969	0.998	0.87	0.222	**0.933**				
IC	0.925	0.93	0.657	0.278	0.642	**0.811**			
MV	0.951	0.981	0.741	0.342	0.326	0.130	**0.861**		
M	0.882	0.889	0.685	0.529	0.755	0.623	0.654	**0.828**	
VS	0.898	0.917	0.62	0.614	0.655	0.524	0.318	0.703	**0.788**

**Table 3:** HTMT discriminant validity.

	**AN**	**CL**	**IC**	**MV**	**M**	**VS**
AN						
CL	0.218					
IC	0.312	0.657				
MV	0.345	0.356	0.238			
M	0.570	0.772	0.672	0.678		
VS	0.627	0.738	0.572	0.367	0.765	

**Fig 2:**
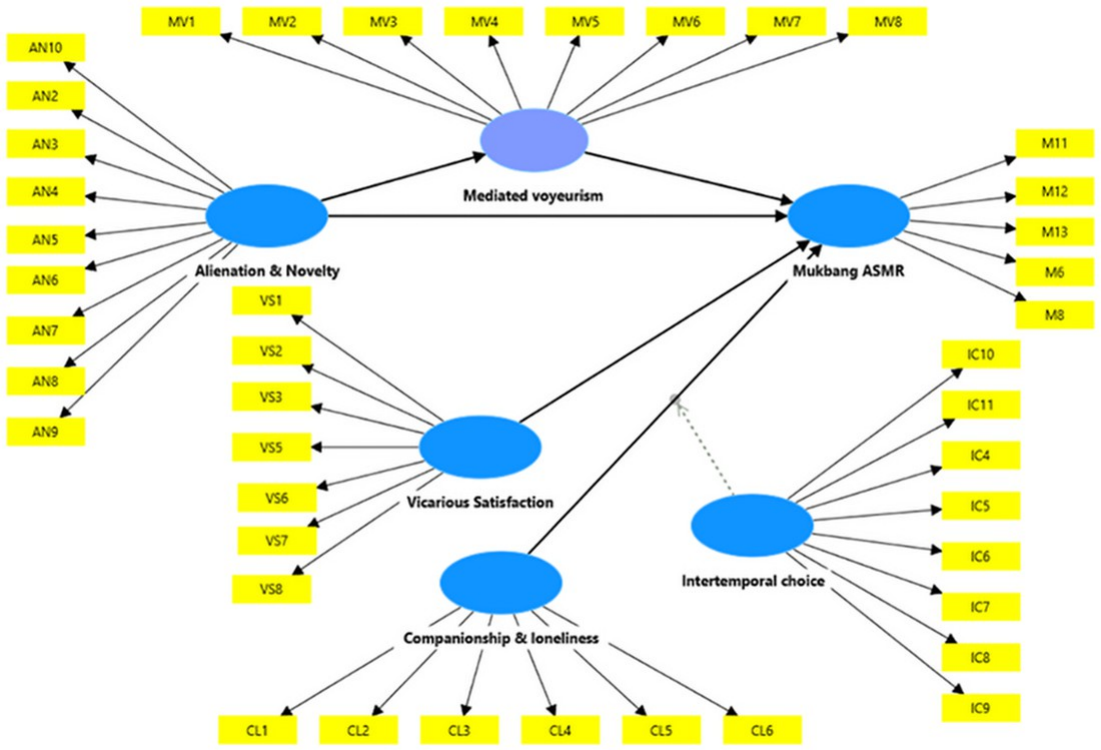
Mukbang ASMR model.

All hypotheses are significant in the predicted direction, with no negative error variance observed for any variable (Table 4 and Fig 2). Mukbang ASMR is positively influenced by vicarious satisfaction (0.116, P = 0.001, T = 3.254, H1), alienation and novelty (0.175, P<0.001, T = 5.375, H2), and companionship and loneliness (0.248, P<0.001, T = 6.483, H3). Among these three exogenous predictors, companionship and loneliness have the most significant impact on Mukbang ASMR (0.248, T = 6.483), where a one-unit increase in companionship and loneliness results in a 0.248 unit increase in Mukbang ASMR obsession, suggesting that social isolation is the primary reasons for Mukbang ASMR obsession among university students. The proposed structural model demonstrates a good fit with a substantial predictive accuracy: Q² = 0.720 (>0), R² = 86.80%, Chi-square = 25175.926, and MAE = 0.461. The model explains 86.80% of the variance in Mukbang ASMR although there are only three exogenous constructs (AN, VS, CL) and one endogenous variable (mediated voyeurism, MV). Alienation and novelty accounts for approximately 11.70% of the variance in mediated voyeurism.

**Table 4:** Structural regression weight (n = 408).

Original sample		STDEV	T Values	P Values	CI 2.5%	CI 97.5%	H	Status
AN -> MV	0.342	0.037	9.195	0.000	0.268	0.414	H4a	Supported
AN -> M	0.175	0.033	5.375	0.000	0.107	0.235	H2	Supported
CL -> M	0.248	0.038	6.483	0.000	0.169	0.320	H3	Supported
MV -> M	0.374	0.021	17.418	0.000	0.333	0.417	H4b	Supported
VS -> M	0.116	0.035	3.254	0.001	0.053	0.190	H1	Supported

This study adopts the default bootstrapping setting (5000 samples) to test the mediation effect of mediated voyeurism between novelty and Mukbang ASMR (H4). Bootstrapping generates empirical confidence intervals (95% CI) through computer-intensive resampling to assess inferences [63]. By resampling, bootstrapping addresses the issue of lower power modeling [64] and resolves concerns regarding the distribution of indirect effect. Research results indicate that mediated voyeurism positively mediates the impact of alienation and novelty on Mukbang ASMR (0.128, T = 9.546, p<0.001, H4). This is a case of partial mediation (Table 5), as the direct effect is also statistically significant (0.175, T = 5.375, p<0.001). The indirect effect (AN→MV→M) accounts for 42.24% of the total effect (0.303, T = 8.959, p<0.001), while the direct effect (AN→M) accounts for 57.76%. The direct effect of alienation and novelty has a slightly greater impact on Mukbang ASMR (57.76%) compared to the indirect effect of mediated voyeurism (42.24%).

**Table 5:** Mediation analysis.

**Indirect Effect**	Original Sample (O)	T Value	P-Value	CI 2.5%	CI 97.5%	Mediation Effect
AN—MV—M (H4)	0.128	9.546	<0.001	0.102	0.155	Partial Mediation (42.24%)
**Direct Effect**	Original Sample (O)	T Value	P-Value	CI 2.5%	CI 97.5%	
AN—M	0.175	5.375	<0.001	0.268	0.414	57.76%
**Total Effect**	Original Sample (O)	TValue	P-Value	CI 2.5%	CI 97.5%	
AN -> M	0.303	8.959	<0.001	0.233	0.366	

**Fig 3:**
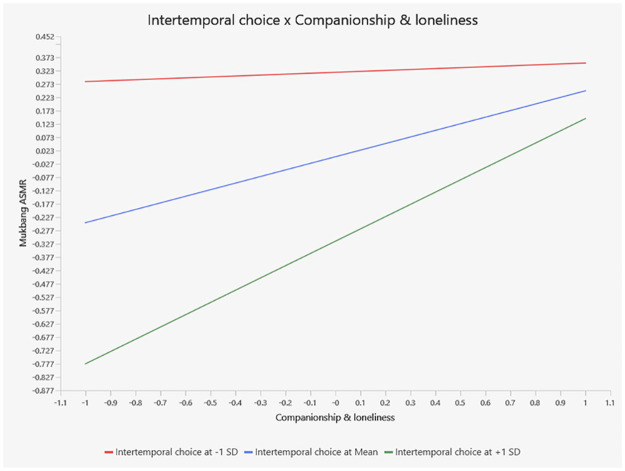
Moderation plot.

A moderator specifies the conditions under which a given effect occurs, as well as the condition under which the direction, nature or strength of an effect varies. This study assumes that the moderation effect of intertemporal choice could be presented as an interaction between companionship and loneliness and mukbang ASMR (H5). Among all three determinants, companionship and loneliness is the most effective factor (0.248, T = 6.483, p<0.001) impacting mukbang ASMR. The intertemporal concept addresses the time perspective notion and suggests that people who evaluate future outcomes as less important and far from happening are more likely to sacrifice that future for sooner joy. Seeking companionship could be the priority for lonely and isolated audiences; hence they may value the immediate feeling/joy (e.g. relief of social isolation) by discounting their future health and lifestyle. This study assumes that intertemporal choice interacting with companionship and loneliness in such a way as to have an impact on the relation toward mukbang ASMR: the relationship could be stronger with the increased impact of intertemporal choice (e.g., discount future more), while a weaker correlation emerging with reduced impact of intertemporal choice (e.g., discount future less). Research findings support the above hypothesis and indicate that intertemporal choice positively moderates the relationship between companionship and loneliness and mukbang ASMR (0.213, T = 4.224, P<0.001, H4). The interaction effect is presented by the moderation plot in Fig 3.

## Conclusion and implications

Understanding the motives of mukbang ASMR is crucial in solving and preventing its harmful consequences on audiences. Theoretically, this study investigates mukbang ASMR by integrating Uses and Gratifications (UG) Theory and Compensatory Internet Use Model (CIUM) from media consumption and psychological perspectives. The proposed Mukbang ASMR model (Fig 2) acts as a mechanism and insights into how the specific antecedents influence Mukbang ASMR obsession. The mediator effect of mediated voyeurism and the moderation effect of intertemporal choice specify particular relationships among key determinants. Research findings indicate that mukbang ASMR is significantly influenced by novelty, vicarious satisfaction, and loneliness. Loneliness is the most significant determinant impacting mukbang ASMR. Mediated voyeurism mediates the impact of alienation and novelty toward mukbang ASMR; and intertemporal choice interacts the relationship between loneliness and mukbang ASMR.

Different from the previous literature, in this study vicarious satisfaction just slightly influences (0.116, T = 3.254) the mukbang ASMR among university students. Most participants do not have their meals together with the social media mukbangers in front of the screens (Table 1). And not every audience feels starving or prefers the exact meal consumed by mukbangers. Hence, the compensation role of vicarious satisfaction on students may not be that great to trigger a vicarious stimulus or a kind of memory in their real life. In fact, most university students who study in other states or countries normally miss the taste of meals prepared by their families, called the ‘taste of hometown’. The timing could be another potential reason why the effectiveness of vicarious satisfaction is less effective in this study. Data collection was conducted in October-November 2022, 6 months after the official announcement of endemic phase by the Malaysian government. After 01 April 2022, all types of national pandemic movement control orders (MCO) and conditional movement control have been taken off in Malaysia. It may partially reduce the impact of vicarious satisfaction of physical or psychological desire for certain food and meal. In addition, this study extends the compensatory internet use model (CIUM) beyond the scope of internet usage by involving non-compensation-oriented antecedents, such as alienation/novelty, and mediated voyeurism in addressing specific suppressed desires. Few studies mention a mild positive effect of vicarious satisfaction on body image or clean eating behavior [9, 65], primarily focusing on female participants with weight concerns. Many participants express dissatisfaction with their own body image, prefer a low-calorie diet, or desire a thinner body [66]. Vicarious pleasure might satisfy their appetite desires, potentially leading to feelings of fullness or reduced food consumption. However, experimental studies indicate that vicarious consumption does not have such an impact [42, 67]. In the context of mukbang ASMR, watching a slim female mukbanger consume large quantities of food or high-calorie dishes can trigger curiosity or frustration regarding viewers’ own diet plans and weight management. This might cause them to question their capability and efficiency in weight management and fitness, potentially drawing them into continued viewing of mukbang ASMR. It’s important to note that not all mukbang ASMR accurately reflects reality in food consumption [68]. The food shown in video may be chewed in front of the camera but then induced to vomit off-screen by mukbangers. The mukbang video content can be edited too, and the mukbanger may not consume the exact amount of food presented on screen. This practice can mislead the public, encourage mukbang obsession, and promote unhealthy consumption norms, poor table manners, and food waste. Society should promote a healthy lifestyle and raise awareness about the potential harmful consequences. Social media platforms and digital communication should be further monitored and regulated by the government or policymakers.

Alienation and novelty emerge as significant predictors (0.175, T = 5.375), positively influencing Mukbang ASMR engagement. This finding aligns with the theory of novelty-seeking and echoes other scholars’ research findings [42, 45], highlighting novelty as an effective rationale for the appeal of food porn in social media. The unique presentation, ways of cooking, and food consumption in mukbang ASMR attract new audiences while existing viewers remain engaged by continuously seeking novel stimuli, such as ’reality show’ or glimpsing into the mukbanger’s true character and daily life. Mediated voyeurism appeals as a mediator in this study, although its independent impact on mukbang ASMR has been supported in other studies [15, 16]. In this study, the indirect effect of mediated voyeurism (0.128, T = 9.546) explains approximately 42.24% variance of mukbang ASMR and the direct effect of alienation and novelty (0.175, T = 5.375) justifies around 57.76% variance of mukbang ASMR. Although some female mukbangers may receive virtual currency (e.g. bonuses or gifts) via live-stream food broadcasting, mediated voyeurism is not the dominant and direct motive in mukbang ASMR obsession. As shown in this study, the direct impact of alienation and novelty is greater (57.76%) on mukbang ASMR than the indirect effect of mediated voyeurism (42.24%). If assessing the impact of alienation/novelty and mediated voyeurism toward mukbang ASMR separately: the former impact is more food-oriented and mainly emphasizes the curiosity of the food, such as raw/spicy food, consuming amount, ways of cooking/exhibition or eating competition. While the later impact is more privacy-oriented and primarily fetishized by peeping into other’s personal lives and confidentiality. The studies of mediated voyeurism about food broadcasting ASMR mainly spotlight female mukbangers only, especially those females with cute faces and sexy outfits. Apart from the previous studies [35, 56], viewing mukbang ASMR is different from watching pornography videos and the specific stimulus (e.g., food vs porn) varies too. Although both Mukbang ASMR (food porn) and pornography may induce strong responses from audiences, they are two different concepts. Food porn refers to visual aesthetics typically embedded with cultural differences [24] and focuses on the pleasurable sensual dimension of food [23], such as the way to cook, present and consume the food. It emerges and arises in the context of the digital age but has been metaphorically associated with gender, body, and sexuality. Audiences, typically male, may fetishize watching mukbang broadcasted by pretty and sexy female mukbangers. However, the voyeuristic gaze in Mukbang ASMR is distinct from the explicit sexual associations found in pornography. Research findings indicate that the pleasant, novelty-driven sensations generated by mukbang viewing provide joy and relaxation, but food-related uniqueness and distinctiveness bring mukbang viewers more gratification and excitement, rather than the mediated sexual desire concealed behind the screens. Mukbang obsession is also linked to harmful consequences and problematic behaviors, including overconsumption, eating disorders, and obesity. Vulnerable individuals may be particularly at risk for these unhealthy eating practices. Social media platforms should be fully aware of the problematic behaviour. Institutions and society are encouraged to promote proper dining manners, healthy lifestyles, and a more harmonious community.

In this research, loneliness emerges as the most significant determinant of mukbang ASMR obsession (0.248, T = 6.483). Previous studies have also highlighted the influence of loneliness on mukbang viewing [15] and social network usage [69]. In this study, loneliness is typically represented in the CIUM model as a notable deficiency in social connection. Individuals seek to compensate for this deficiency by pursuing a sense of belonging, social support, and self-affirmation through viewing mukbang ASMR and interacting with mukbangers. According to Uses and Gratifications (UG) theory, the gratification perceived from image or video-based content (e.g., food broadcasting) on social media cues the ‘realism heuristic’ [70]. The feeling of companionship appears more realistic and believable. Enhanced by the ASMR property, audiences experience greater and more pleasant relief from social isolation. However, this feeling of companionship is only temporary, as mukbang serves merely as a channel/tool to relieve perceived loneliness. Mukbang cannot fundamentally solve loneliness as the root of loneliness is more related to psychological well-being and beyond the scope of mukbang ASMR. Institutions and communities should pay more attention to mental care, personal disposition, and desire for commensality among the younger generation. Additionally, intertemporal choice positively moderates the relationship between loneliness and mukbang ASMR (0.213, T = 4.224), implying that lonely audiences are keen to seek immediate relief from isolation, often trading long-term social well-being for instant gratification. They favor immediate pleasure and sooner rewards over long-term benefits. This tendency reflects a risky pattern in their decision-making. Institutions and society should offer appropriate valuation guidance and cultivate proper social-life norms. A summary of stakeholder implications and recommended interventions is presented in Table 6.

**Table 6:** Implications and intervention.

Stakeholders	Research findings & LR highlights	Implication & Intervention
Policymaker & communities	i). Antecedents of Mukbang ASMR obsession ii). Consequence of Mukbang, and roles of mediation and moderation	i). Regulate social media platforms and monitor digital media communications. ii). Raise awareness of harmful consequences and encourage healthy social well-being
Institutions & society	i). Loneliness has a greater influence on Mukbang ASMR obsession. Isolated individuals seek instant relief and gratification and are more obsessive in mukbang ASMR. ii). Embedding with visual esthetics, food porn carries a distinguished interpretation.	i). Create a welcoming and diverse learning environment and establish a peer mentoring program to enhance students’ social engagement. ii). Provide psychological counseling and mental health seminars to reduce perceived loneliness and address mental health issues. iii). Offer academic guidance on future valuation and understanding of contemporary terms, such as "food porn".
Viewers	i). Mukbang ASMR motives and potential harmful consequences	i) Understand mukbang motives and potential consequences and moderate exposure to Mukbang ASMR ii) Incorporate various social activities and pursue healthy lifestyle.

Given the cross-sectional design of this study, the primary data collection spanned four weeks and targeted university students only. This research cannot conclude a long-term effect of key motives on mukbang ASMR obsession. The closed-ended survey questions limit respondents to predefined options, which might not fully capture their true opinions or experiences. The future study could consider adopting a longitudinal qualitative study to further explore the underlying insights of mukbang ASMR from various perspectives, including gender, region, type of food, and social media platforms.

## Supporting information

S1 Appendix Measurement scales and factor loadings. (DOCX)

S2 Appendix Key dimensions findings. (DOCX)
