# Supplementary material for: Why am I obsessed with viewing mukbang ASMR? The roles of mediated voyeurism and intertemporal choice
Source: PLoS One. 2024 Sep 19;19(9):e0308549. doi: 10.1371/journal.pone.0308549 (PMC11412535; doi:10.1371/journal.pone.0308549)
Supplement: S1 Appendix — (DOCX) [file pone.0308549.s001.docx]

| **Appendix 1: Measurement Scales and Factor Loadings** | **Items** | **Factor Loading** |
| --- | --- | --- |
| **Mukbang ASMR (M)** |  |  |
| M6. I like to hear the sound of biting, chewing, and crunching in the Mukbang ASMR videos. | M6 | 0.886 |
| M8. I may share my thoughts, experiences, the mood of the day, feelings, etc. during the Mukbang ASMR live-streaming. | M8 | 0.695 |
| M11. I may interact with the host or other viewers when I am watching Mukbang ASMR live-streaming.  M12. I like to see the mukbanger eat various types of food.  M13. I more likely watch Mukbang ASMR videos when I am interested in a certain type of food.  Source: [15] Pereira et al (2019) & [12] Anjani et al (2020) | M11  M12  M13 | 0.779  0.865  0.897 |
| **Vicarious Consumption (VC)** |  |  |
| VS1. I watch mukbang because it gives me a sense that I am the one who eats.  VS2. I watch mukbang because I enjoy seeing others eating food. | VS1  VS2 | 0.841  0.813 |
| VS.3 I watch mukbang because the sound of chewing and biting can provide a satisfying sentiment. | VS3 | 0710 |
| VS.5 I watch mukbang to satisfy my cravings for different food that I am not allowed to eat.  VS6. I watch mukbang because it can vicarious me to try food from other countries or food that unable to buy locally.  VS.7. I watch mukbang to have virtual satisfaction of eating.  VS.8. I watch mukbang to avoid actual eating and consuming when I am on diet.  Source: [39] Kircaburun et al (2021) & [37] Song et al (2023) | VS5  VS6  VS7  VS8 | 0.753  0.766  0.885  0.731 |
| **Alienation & Novelty (AN)** |  |  |
| AN2 I watch mukbang to discover novel foods from different cultures.  AN3 I’m able to know various foods and cooking methods by watching mukbang  AN4. I watch mukbang because it is more creative than other content. | AN2  AN3  AN4 | 0.880  0.866  0.845 |
| AN5. Watching mukbang content videos is new and refreshing.  AN6. The content of mukbang creates a novel environment on social media.  AN7. The content of unscripted shows (e.g. mukbang) is more interesting than scripted shows.  AN8. I enjoyed watching unscripted shows (e.g. mukbang) more than scripted shows (storyline-based videos).  AN9. I watch mukbang because I like seeing real people face challenging situations.  AN10. I watch mukbang because I like it when something unexpected occurs in the live streaming or videos.  Source: [45] Assaker et al (2011); [43] Sung et al (2016)  **Mediated Voyeurism (MV)** | AN5  AN6  AN7  AN8  AN9  AN10 | 0.834  0.870  0.833  0.799  0.736  0.908 |
| MV1. I watch mukbang because I find the character attractive and private. | MV1 | 0.909 |
| MV2. I enjoyed watching mukbang videos that help me get an observation into people’s private moments.  MV3. I like mukbang content that shows a side of people that I would not normally see.  MV4. I enjoy watching mukbang videos that provide access to things that people try to hide. | MV2  MV3  MV4 | 0.772  0.901  0.864 |
| MV5. I enjoy the mukbang content videos because you never know what might see from the content.  MV6. I get satisfaction out of watching mukbang when they are unaware of the purpose of watching.  MV7. I watch mukbang because I like to hear my favorite mukbanger describe his or her feelings and recount stories or daily lifestyle about him or herself.  MV8. I watch mukbang because it can provide me with the inspiration to get a different lifestyle.  Source: [55] Baruh (2010) & [52] Park et al (2022)  **Companionship & Loneliness (CL)** | MV5  MV6  MV7  MV8 | 0.886  0.872  0.943  0.713 |
| CL1. I watch mukbang to feel less lonely.  CL2. I watch mukbang because I lack companionship.  CL3. I watch mukbang because I can find a kind of companionship.  CL4. I watch mukbang because there are people, I feel close to.  CL5. I watch mukbang because there are people I can talk to.  CL6. I watch mukbang videos when I am eating alone and all by myself.  Source: [7] Kircaburun, et al (2022) & [48] Wang at al (2021)  **Intertemporal Choice (IC)**  **RM 10,000 Iteration**  IC4. Imagine you have been awarded a RM 10,000 prize that you will receive in 6 weeks. How much of that prize would you be willing to give up to have it today?  Slider scale of RM0 to RM9,999  IC5. Now imagine you have been awarded a RM10,000 prize that you will receive in 9 months. How much of that prize would you be willing to give up to have it today?  Slider scale of RM0 to RM9,999  IC6. Finally, imagine the same situation but that you are offered RM10,000 prize that you have to wait 3 and a half years to receive. How much of that prize would you be willing to give up to have it today?  Slider scale of RM0 to RM9,999  **RM100,000 Iteration**  IC7. Imagine you have been awarded a RM100,000 prize that you will receive in 6 weeks. How much of that prize would you be willing to give up to have it today?  slider scale of RM0 to RM99,999  IC8. Now imagine you have been awarded a RM100,000 prize that you will receive in 9 months. How much of that prize would you be willing to give up to have it today?  slider scale of RM0 to RM99,999  IC9. Finally, imagine the same situation but that you are offered RM100,000 prize that you have to wait 3 and a half years to receive. How much of that prize would you be willing to give up to have it today?  Slider scale of RM0 to RM99,999  Non-financial Iteration  IC10. Imagine you are offered 10 Grab food vouchers this year. How many extra food vouchers would you like to have to wait until next year to have those extra Grab vouchers?  Slider scale 0-21 vouchers  IC11. Imagine you are offered 10 free Starbucks coffee this year. How many extra free coffees would you like to have to wait until next year to have those extra Starbucks coffee?  Slider scale of 0-35 coffee  Source: [32] Kennedy (2020) | CL1  CL2  CL3  CL4  CL5  CL6  IC4  IC5  IC6    IC7  IC8  IC9  IC10  IC11 | 0.979  0.979  0.976  0.972  0.976  0.674  0.710  0.785  0.674  0.848  0.872  0.735  0.850  0.769 |
|  |  |  |
