# Supplementary material for: Why am I obsessed with viewing mukbang ASMR? The roles of mediated voyeurism and intertemporal choice
Source: PLoS One. 2024 Sep 19;19(9):e0308549. doi: 10.1371/journal.pone.0308549 (PMC11412535; doi:10.1371/journal.pone.0308549)
Supplement: S2 Appendix — (DOCX) [file pone.0308549.s002.docx]

**Appendix 2**: Key Dimensions and Findings

| **Key Findings & Authors** | **VS** | **AN** | **MH** | **CL** | **SMC** | **Mukbang** | **MV** | **IC** |
| --- | --- | --- | --- | --- | --- | --- | --- | --- |
| Vicarious satisfaction and attractiveness of content performers had an effect on attitudes toward mukbang. Perceived ease of use and attitude toward mukbang content had an impact on the intention to watch mukbang content [37].  Title: Understanding Social Media Users' Mukbang Content Watching: Integrating TAM and ECM  Author(s): Song (2023) | ● |  |  |  |  |  |  |  |
| Vicarious satisfaction, enjoyment, and information influenced the intention to watch mukbang via attitudes toward mukbang. Exposure and attractiveness had an impact on the intention to watch mukbangs via para-social relationships [40].  Title: How Attitude and Para-Social Interaction Influence Purchase Intentions of Mukbang Users: A Mixed-Method Study  Author(s): Song et al (2023) | ● |  |  |  |  |  |  |  |
| Asians tend to watch Mukbang due to host attractiveness and social normative influence, while Caucasians watch such shows due to host attractiveness, perceived novelty and social normative influence [15].  Title: I like watching other people eat: A cross-cultural analysis of the antecedents of attitudes towards Mukbang  Author(s): Pereira et al (2019) | ● | ● |  | ● |  |  |  |  |
| Both novelty seeking and low satisfaction among travelers temper immediate intent to return. Conversely, a positive image of the destination enhances both immediate and future intentions to return [45].  Title: Examining the effect of novelty seeking, satisfaction, and destination image on tourists' return pattern: A two factor, non-linear latent growth model  Author(s): Assaker et al (2011) |  | ● |  |  |  |  |  |  |
| The ability of exposure to docusoap reality television as well as its perceived reality to predict normative beliefs about aggression, even under multiple controls [44].  Title: Is Reality TV a Bad Girls Club? Television Use, Docusoap Reality Television Viewing, and the Cultivation of the Approval of Aggression  Author(s): Scharrer & Blackburn (2018) |  | ● |  |  |  |  |  |  |
| South Koreans tend to use media more frequently during meals than Germans, especially for social purposes. Younger age only predicts meal-concurrent media use in the German sample [42].  Title: Investigating meal-concurrent media use: Social and dispositional predictors, intercultural differences, and the novel media phenomenon of mukbang eating broadcasts  Author(s): Stein & Yeo (2021) |  | ● |  |  |  |  |  |  |
| Mukbang watching may be problematic for a minority of emerging adults and that problematic mukbang watching warrants further examination of its impact on mental health and well-being [39].  Title: Problematic Mukbang Watching and Its Relationship to Disordered Eating and Internet Addiction: A Pilot Study Among Emerging Adult Mukbang Watchers  Author(s): Kircaburun et al (2021) |  |  | ● |  |  |  |  |  |
| Problematic mukbang watching by developing and validating the Mukbang Addiction Scale (MAS). Individuals spending lots of time watching mukbang could lead to the addictive use of that activity. Assessment and clinical-related implications of the findings are illustrated in accordance with other excessive behaviours [29].  Title: Development and Validation of the Mukbang Addiction Scale  Author(s): Kircaburun et al (2021) |  |  | ● |  |  | ● |  |  |
| Problematic YouTube use PMW was positively related to loneliness and PYU. Depression was positively and directly associated with PYU but was not associated with PMW [6].  Title: Compensatory Usage of the Internet: The Case of Mukbang Watching on YouTube  Author(s): Kircaburun et al (2021) |  |  | ● | ● |  |  |  |  |
| Results from the scoping review indicated that viewers use mukbang watching for social reasons, sexual reasons, entertainment, eating reasons, and/or as an escapist compensatory strategy. Furthermore, mukbang watching appears to have both beneficial consequences (e.g., diminishing feelings of loneliness and social isolation, constructing a virtual social community,) and non-beneficial consequences (e.g., altering food preferences, eating habits, and table manners, promoting disordered eating, potential excess, and 'addiction' [16].  Title: The Psychology of Mukbang Watching: A Scoping Review of the Academic and Non-academic Literature  Author(s): Kircaburun et al (2021) |  |  | ● | ● |  | ● |  |  |
| The intersections with health were observed from impacts on family/community engagement, culinary skills development, and mental health and eating habits.These practices also structured specific social interactions, such as virtual food communities and commensality, during the COVID-19 pandemic [19].  Title: Digital Forms of Commensality in the 21st Century: A Scoping Review  Author(s): Pereira-Castro et al (2022) |  |  | ● |  |  |  |  |  |
| Two overarching themes were identified from Youtube and Reddit posts: a viewer perspective, by which users discuss mukbang without describing any personal involvement, and a participant perspective, by which users describe their own experiences of affects and behaviors in response to watching mukbang [47].  Title: Mukbang and Disordered Eating: A Netnographic Analysis of Online Eating Broadcasts  Author(s): Strand & Gustafsson (2020) |  |  |  | ● |  |  |  |  |
| Perceived loneliness to be reduced by Mukbang-based or in-person commensality, but they were also aware of the risks of enhancing food intake and/or being shifted toward less healthy food choices in these two scenarios. Participants expected cloud-based commensality to provide the benefits of reducing loneliness without the health-compromising risks of increasing food intake or unhealthy eating [48].  Title: Cloud-Based Commensality: Enjoy the Company of Co-diners Without Social Facilitation of Eating  Author(s): Wang et al (2021) |  |  |  | ● |  |  |  |  |
| A cross-sectional study find that channel factors (food product offerings and convenience) and influencer factors (credibility and parasocial relationship) significantly enhance consumers' perceived value, leading to impulse purchase and overconsumption [20].  Title: The Impact of Mukbang Live Streaming Commerce on Consumers' Overconsumption Behavior  Author(s): Lee & Wan (2023) |  |  |  |  | ● |  |  |  |
| Systematic literature review with PRISMA indicates that positive relationships between exposure to certain material on social media (e.g., fast-food advertisements, clean eating, eating videos) and body image concerns, disordered eating, and consumption of, or a willingness to consume the foods seen on social media; however, studies that focused on 'healthy food' content showed no such association [9].  Title: Digging into digital buffets: A systematic review of eating-related social media content and its relationship with body image and eating behaviours  Author(s): Wu et al (2024) |  |  |  |  | ● |  |  |  |
| Study investigates social media exposure impact on food choices among South Korean adolescents (male and female groups). Both sexes were more likely to frequently eat unhealthy food than students who did not watch eating shows. Food broadcasting significantly influences adolescents' food choices, highlighting the need for interest in emerging cultures and corresponding health policies [10].  Title: Association between watching eating shows and unhealthy food consumption in Korean adolescents  Author(s): Joo et al (2024) |  |  |  |  | ● |  |  |  |
| Research explores the ethical controversy of mukbang live streams and analyzes their gender politics by parsing the mystifying image of female 'big stomach kings', whose slim bodies are in stark contrast to their enormous appetites [68].  Title: Waste on the Tip of the Tongue: Social Eating Livestreams (Chibo) in the Age of Chinese Affluence  Author(s): Qu (2021) |  |  |  |  | ● |  |  |  |
| The study explores the predictive role of problematic mukbang watching on adverse consequences and suggests that mukbang watching may be problematic for a minority of emerging adults [39].  Title: Problematic Mukbang Watching and Its Relationship to Disordered Eating and Internet Addiction: A Pilot Study Among Emerging Adult Mukbang Watchers  Author(s): Kircaburun et al (2021) |  |  |  |  |  | ● |  |  |
| A mixed-methods study of viewers on their practices and motivations around watching mukbang-video streams of people eating large quantities of food. Viewers' experiences provide insight on future technologies for multisensorial video streams and technology-supported commensality [12].  Title: Why do people watch others eat? An Empirical Study on the Motivations and Practices of Mukbang Viewers  Author(s): Anjani et al (2020) |  |  |  |  |  | ● |  |  |
| Voyeurism, content production, and content consumption have direct effects on intensity of SNS use. Exhibitionism and voyeurism have indirect effects on intensity of SNS use through content production and content consumption [52].  Title: How exhibitionism and voyeurism contribute to engagement in SNS use: The mediating effects of content production and consumption  Author(s): Park et al (2022) |  |  |  |  |  |  | ● |  |
| Media commentators have frequently argued that the rising popularity of reality programs stems from the show's ability to accommodate television viewers' voyeuristic needs. There exists a positive relationship between voyeurism and consumption of reality programming. Trait voyeurism is negatively related to the consumption of fictional programming [55].  Title: Mediated Voyeurism and the Guilty Pleasure of Consuming Reality Television  Author(s): Baruh (2010) |  |  |  |  |  |  | ● |  |
| Sociosexuality and sexual compulsivity predict sex differences in voyeuristic interest in the population. There were clear sex differences in voyeuristic and exhibitionistic repulsion that were partially mediated by the serial combination of sociosexuality and sexual compulsivity. Coupled with an evolutionary perspective, the role sociosexuality plays in voyeuristic and exhibitionistic interest [54].  Title: Sex Differences in Voyeuristic and Exhibitionistic Interests: Exploring the Mediating Roles of Sociosexuality and Sexual Compulsivity from an Evolutionary Perspective  Author(s): Thomas et al (2021) |  |  |  |  |  |  | ● |  |
| Mediation analyses show that time perception mediates a significant portion of cognitive load's effect on intertemporal choices. Study demonstrates that time preference identified by intertemporal choices might be confounded by potentially biased time perception, calling for improving suboptimal time-related economic decisions due to an individual's misperception of how time flies [30].  Title: How time flies: Time perception and intertemporal choice  Author(s): Chen et al (2024) |  |  |  |  |  |  |  | ● |
| The pattern of intertemporal preferences is related to critical behavioural aspects involving individuals' emotional and cognitive spheres. The characteristics of the discount function can provide a quantitative interpretation of pathological conditions [31].  Title: An original approach to anomalies in intertemporal choices through functional data analysis: Theory and application for the study of Hikikomori syndrome  Author(s): Ventre et al (2024) |  |  |  |  |  |  |  | ● |
| Empirical evidence shows that people with better self-control to a greater extent have the self-regulatory ability to act in line with their long-term goals. The relationship between self-control and self-regulatory behavior was investigated both directly and indirectly, i.e., through affective forecasting ability. This is of great interest as it is necessary to forecast one's emotional response to future events to make choices that maximize one's happiness [51].  Title: Forecasting emotions: exploring the relationship between self-control, affective forecasting, and self-regulatory behavior  Author(s): Strömbäck (2024) |  |  |  |  |  |  |  | ● |
| Study explores the role of temporal discounting—the extent to which people devalue future rewards—in people’s reports of their subjective well-being This study evidences on the relationship between discount rates and evaluative and hedonic subjective well-being. Results suggest that people who devalue future rewards are less satisfied with life and less happy than those who place greater value on future outcomes. However, those who discount heavily are also more likely to expect that they will be happier in the future [32].  Title: Subjective wellbeing and the discount rate  Author(s): Kennedy (2020) |  |  |  |  |  |  |  | ● |
| ** Dimension Abbreviation:  **VS:** Vicarious Satisfaction **AN**: Alienation & Novelty  **MH**: Mental Health **CL**: Companionship & Loneliness  **SMC**: Social Media Consumption **Mukbang**: Mukbang ASMR  **MV**: Mediated Voyeurism **IC**: Intertemporal Choice |  |  |  |  |  |  |  |  |
